# Three developed spectrophotometric methods for determination of a mixture of ofloxacin and ornidazole; application of greenness assessment tools

**DOI:** 10.1186/s13065-023-00932-3

**Published:** 2023-03-13

**Authors:** Khadiga M. Kelani, Asmaa G. Gad, Yasmin M. Fayez, Amr M. Mahmoud, Ahmed M. Abdel-Raoof

**Affiliations:** 1 Analytical Chemistry Department, Faculty of Pharmacy, Cairo University, El-Kasr El-Aini Street, Cairo, 11562 Egypt; 2 Analytical Chemistry Department, Faculty of Pharmacy, Modern University for Technology and Information, Cairo, Egypt; 3 Pharmaceutical Analytical Chemistry Department, Faculty of Pharmacy, Al-Azhar University, Nasr City, Cairo, 11751 Egypt

**Keywords:** Green analytical chemistry, Ratio difference, Mean centering of ratio spectra, Continuous wavelet transform of ratio spectra

## Abstract

This work is dedicated to the greenness estimation of three proposed spectrophotometric techniques [e.g., ratio difference (RD), mean centering of ratio spectra (MCR) and continuous wavelet transform of ratio spectra (CWT)] for the determination of a binary combination named Ofloxacin (OFL) and Ornidazole (ORN). Applying the green analytical chemistry methods to assess the proposed methods has widely attained the analytical community care. The greenness assessment was performed via three evaluation approaches; the “Analytical Eco-Scale”, the “National Environmental Method Index” (NEMI) and “Green Analytical Procedure Index” (GAPI). Following the examination of the zero spectrum of OFL and ORN, it is observed that OFL and ORN spectra are overlapped, so they can be detected by the methods mentioned previously. The ratio difference method was carried out at wavelengths of 294.6 nm and 265.6 nm for OFL, 292 nm and 315 nm for ORN. The linear range was (2–15 µg/mL) for OFL and (3–30 µg/mL) for ORN. The MCR method based on the use of mean centered ratio spectra in dual steps and calculating the second ratio spectra mean centered values at 294.6 nm for OFL and 315 nm for ORN. The continuous wavelet transformation which carried out using MATLAB at wavelengths of 265 nm for OFL and 306 for ORN. These techniques were intended for the binary mixture analysis in bulk powder and pharmaceutical formulations with high recoveries. The developed methods were validated according to ICH guidelines. All techniques were statistically compared to either an official method for OFL or a reported method for ORN and the results indicate that there were not any significant differences.

**Supplementary Information:**

The online version contains supplementary material available at 10.1186/s13065-023-00932-3.

## Introduction

**Fig. 1:**
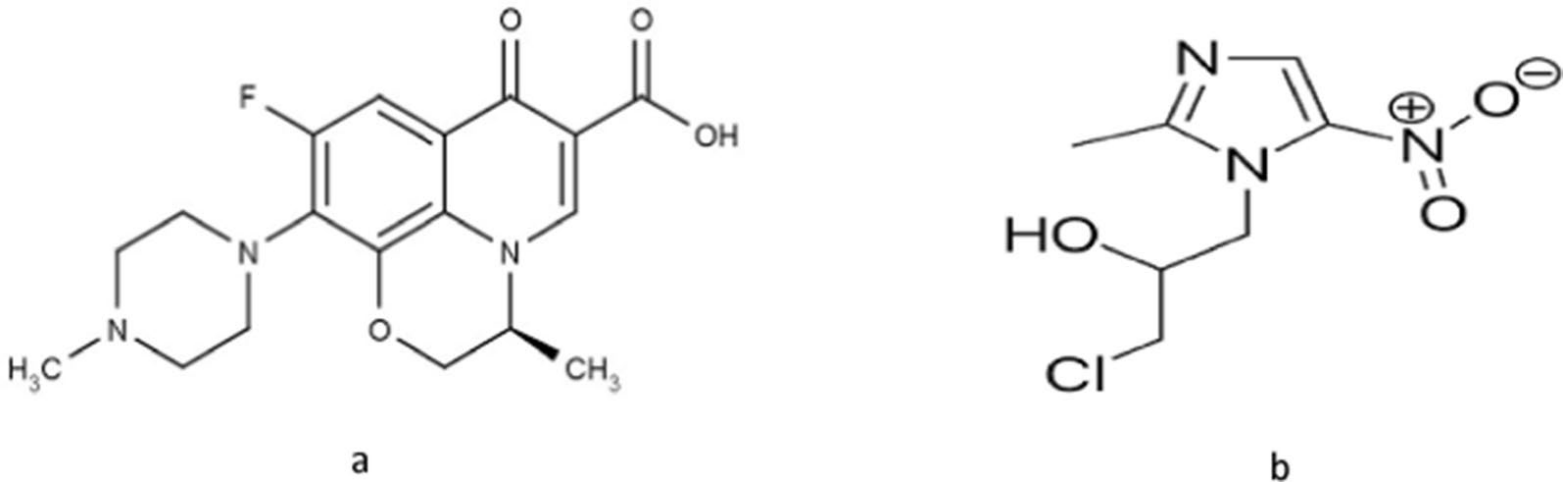
Chemical structure of Ofloxacin **a** and Ornidazole **b**

As stated by World Health Organization (WHO) bacterial infections are the second main cause of worldwide mortality [1]. Accordingly, quality control testing is highly important for the antimicrobial agents. Ofloxacin (OFL), its chemical structure is shown in (Fig. 1a), is a fluoroquinolone antibiotic, that is extremely active in case of gram-positive bacteria and also gram-negative one. Moreover, OFL is active against chlamydia, legionella and mycoplasma [2]. The bactericidal effect of OFL depends on bacterial DNA gyrase inhibition, that enzyme produces a negative supercoil in DNA, allowing the two process of transcription and reproduction to occur [3]. Ornidazole (ORN), its chemical structure is presented in (Fig. 1b), passes into the cell via diffusion where redox proteins, only found in anaerobic organisms, catalyse the reduction of the nitro group into a product that induces a cytotoxic effect via destruction of DNA [4]. OFL and ORN are combined to treat parasitic and microbial infections. The binary mixture is intended for the treatment of gastrointestinal infections, acute diarrhea, gynecological infections, lung and urinary tract infections [5]. There are various reported techniques for the analysis of this binary mixture including: RP-HPLC [6–8], TLC [4], HPTLC [7, 9, 10], capillary zone electrophoresis [11, 12], voltammetry [13], HPLC [7, 14], UPLC tandem mass spectrometry [15] and spectrophotometric methods [16–19]. Although the developed separation methods are sensitive and selective, but these methods commonly use harmful and toxic solvents, therefore, developing green and environmentally friendly methods is necessary to deliver greater benefits to environment and staff.

Three varying spectrophotometric techniques were used for concurrent determination of the studied drugs. RD is an easy, fast, applicable and selective method for estimation of binary mixtures. It benefits from simplicity, minimal data processing and broader range of applications. [20–23]. MCR is a new easy, simple, fast and applicable method for determining components in binary mixtures with overlapping spectra. [21, 22, 24–27]. CWT has been used in analytical chemistry realm since 1992[28] as a signal processing technique. Continuous wavelet transform is a simple, fast, and selective signal processing method for determining components in binary mixtures with overlapping spectra [29]. Its advantages include de-noising, smoothing, and broader range of applications [30–32].

The use of green analytical chemistry (GAC) term is associated frequently to many important conceptions like: environmental impact, maintainable evolution and least waste. The main goal of GAC is to develop green procedures for pharmaceuticals analysis in quality control field. There are twelve principles for application of GAC [33]. These principles are to be assessed by the analyst. To evaluate the greenness of the developed method, some tools were developed that depend mostly on those twelve principles as: Analytical Eco-Scale and National Environmental Methods Index (NEMI). In spectrophotometric approaches, little factors would affect its greenness like the main aspects of miniaturization, sample preparation, use of bio-accumulative or non-green reagents and waste products [34]. The analyst greatest challenge is to make a comparison between the quality and efficiency of his technique with the human being and environmental safety and also the cost of the experiment.

The goal of the study was developing and validating easy, precise, applicable, sensitive, accurate and green three spectrophotometric techniques for selective quantitative determination of OFL and ORN in bulk powder and marketed preparations. The proposed techniques were validated according to the ICH recommendations. The green analytical chemistry concept was used to evaluate the RD, MCR, and CWV methods.

## Experimental

### Instruments

SHIMADZU UV-1650 and 1800 PC spectrophotometer (Kyoto, Japan), double beam UV-visible spectrophotometer.

The UV-PC personal spectroscopy software version (2.21) (Shimadzu, Kyoto, Japan). The wavelength scanning speed was 2800 nm/min, with a spectral band width of 0.2 nm. The PLS toolbox was used to implement the MCR method in MATLAB 8.2.0.701 (R2013b). The wavelet toolbox was used to implement the CWT method in MATLAB 8.2.0.701 (R2013b).

### Samples and chemicals

In this work; the used solvents and chemicals were of analytical purity.

### Standard samples

Standards of OFL and ORN were obtained from National Organization for Drug & Control Research. Their purities were evaluated by applying the official method for OFL and reported method for ORN. and estimated to be 100.4 ± 0.8 for OFL and 99.4% ± 0.9 for ORN.

### Market sample

ORNI-O^™^ tablet was manufactured and purchased from international market (Indian Market), Batch number (ALT19317), it is claimed that each tablet contains both 500 mg ORN and 200 mg OFL.

### Solutions preparation

#### Standard stock solutions of OFL and ORN (1 mg/mL)

To achieve a final concentration of 1 mg/mL, portions of 100 mg each of OFL and ORN were weighed, placed into two 100 mL flasks, and then dissolved in methanol. The stock solutions were kept in the refrigerator for 5 days during the laboratory work period.

#### Working standard solutions of OFL and ORN (0.1 mg/mL)

Two separate 50-mL volumetric flasks were filled with 5-mL of the ORN and OFL from their stock (1 mg/mL). Methanol was used to complete the volume, producing working standard solutions with final concentrations of (0.1 mg/mL).

### Procedure

ICH guidelines were used to validate the developed methods [35].

#### Linearity

From the working standard solutions (0.1 mg/mL), (0.2, 0.4, 0.6, 0.8, 1, 1.2, 1.5 mL) of OFL and (0.3, 0.5, 1, 1.5, 2, 2.5, 3 mL) of ORN were transferred into 10-mLvolumetric flasks and the flasks were completed by methanol to attain these ranges of concentration, (2–15 µg/mL) for OFL and (3–30 µg/mL) for ORN. Both drugs were scanned against methanol as a blank in the 200–400 nm wavelength range.

#### Ratio difference method (RD)

The prepared OFL solutions spectra were recorded with methanol as a blank after that, obtained spectra were divided by (5 µg/mL) ORN spectrum as a divisor. The peak amplitudes difference at (265.6 and 294.6 nm) was then plotted versus the respective OFL concentrations. Subsequently, the regression equation was computed. The solutions of ORN were then recorded with methanol as a blank, and the spectrum of OFL (4 µg/mL) was used as a divisor. After that, the difference in peak amplitudes at (292 and 315 nm) was then plotted against ORN concentrations. The regression equation was then calculated.

#### Mean centering of ratio spectra spectrophotometric method (MCR)

The ratio spectra were mean centered and the amplitude of the mean centered spectra for OFL and ORN was calculated at 296 nm and 315 nm, respectively. The regression equation was computed after constructing a calibration curve between the mean centered value and the concentrations in µg/mL.

#### Continuous wavelet transform of ratio spectra (CWT)

The absorption spectra of the formulated solution were measured using methanol as a blank. After that, divided by the spectrum of (5 µg/mL) ORN as a divisor to determine OFL, and then divided by the spectrum of OFL (4 µg/mL) to determine ORN. The ratio spectra were transformed to the wavelet domain, and the wavelet coefficients were computed using the biorthogonal (bior 2.4) family and [scale value (a) = 25]. The transformed signals’ amplitudes were detected at 285 nm for OFL and 306 nm for ORN. After that, the regression equation was calculated.

#### Laboratory prepared mixtures

Different volumes of OFL and ORN were exactly transferred from their standard working solutions (0.1 mg/mL) into a series of 10-mL flasks and the flasks were completed with methanol to prepare mixtures with varying ratios of the two drugs.

#### Pharmaceutical dosage form

After precisely weighing and finely powdering ten pills, one tablet's average weight was calculated. A precisely measured powder sample of 500 mg for ORN and 200 mg for OFL was transferred to a volumetric flask with a volume of 100 mL, dissolved in 20 mL of methanol, after that sonicated for 20 min. The volume was then topped off with methanol. The solution was subsequently filtered via double-ring filter paper. Following that, the solution filtered was diluted to 25 µg/mL for ORN and 10 µg/mL for OFL.

#### Standard addition technique

The developed methods’ validity was then evaluated via using the standard addition method, in which diverse known concentrations of pure samples were added to a known weight of the marketed drug, which was then examined by the developed methods, and the recovery of the added OFL and ORN was measured from their equivalent regression equations.

## Results and discussion

**Fig. 2:**
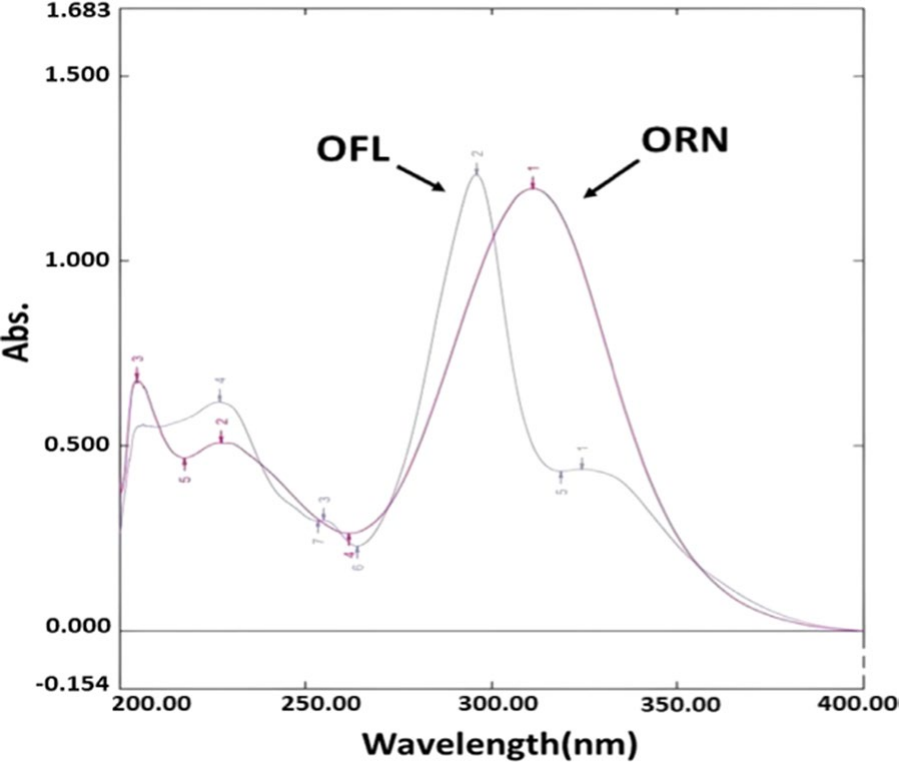
(D^0^) Zero order spectra of 12 µg/mL OFL and 30 µg/mL ORN in methanol

The purpose of this study is to compare the RD, MCR, and CWT methods for the study of binary combinations with severely overlapped spectra. This was accomplished by estimating OFL and ORN simultaneously in both their pure and tablet forms. OFL and ORN had overlapping zero-order absorption spectra (D^0^), as shown in (Fig. 2). Therefore, different spectral manipulating methods, including the before mentioned methods, have been used for determination of OFL and ORN. Finally, the spectrophotometric methods were assessed for greenness using the green analytical chemistry concept.

### Ratio difference method

**Fig. 3:**
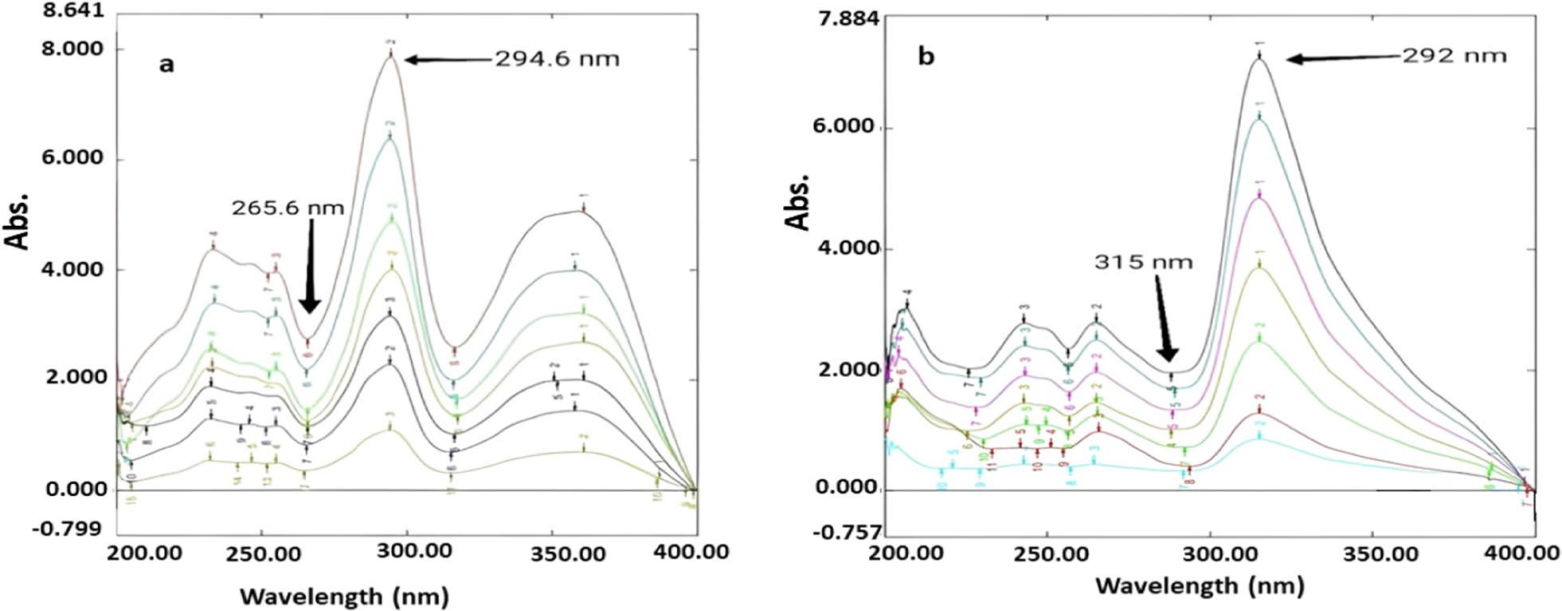
**a**: Division spectra of OFL in concentration range (2–15 µg/mL) and (5 µg/mL) of ORN as a divisor and methanol as a solvent; **b**: Division spectra of ORN in concentration range (3–30 µg/mL) and (4 µg/mL) of OFL as a divisor and methanol as a solvent

RD technique was established to determine OFL and ORN in their combination with minimum data processing, and superior selectivity. Different divisor concentrations of ORN were attempted (5, 10, 15, 20, 25 and 30 µg/mL) and the concentration (5 µg/mL) was observed to be the most preferable concerning minimal noise as shown in (Fig. 3a). Moreover, different divisor concentrations of OFL were tried (2, 4, 6 and 8 µg/mL) and the concentration (4 µg/mL) was noticed to be the most preferable in terms of minimal noise as exposed in (Fig. 3b).

Calibration curves for OFL (Additional file 1: Fig. S1) and ORN (Additional file 1: Fig. S2) were built in relation to the difference in peak amplitude at wavelengths (265.6 and 294.6 nm) and (292 and315 nm), respectively and the concentration ranges of (2–15 µg/mL) and (3–30 µg/mL), correspondingly. The regression equations were computed and appear to be:

Y (265.6 nm—294.6 nm) = 0.3361X + 0.112, r = 0.9999. (For OFL).

Y (292 nm—315 nm) = 0.1751X + 0.0139, r = 0.9998. (For ORN).

Where Y is the difference in peak amplitude, X is the drug concentration in µg/mL and r is the correlation coefficient as shown in Additional file 1: Figs. S1, S2). The developed ratio difference procedure was effectively used to determine OFL and ORN in with varying proportions of OFL and ORN.

### Mean centering of ratio spectra spectrophotometric method (MCR)

**Fig. 4:**
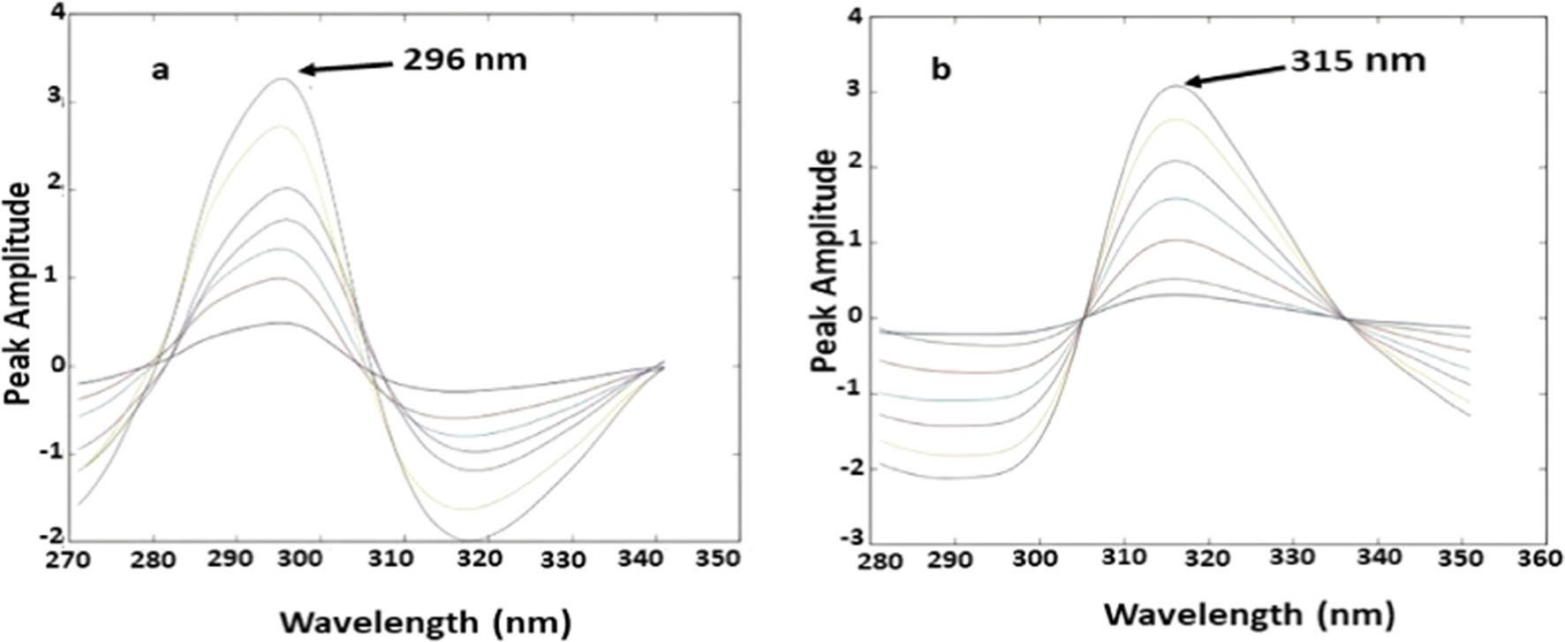
**a**: Mean Centering of the ratio spectra of OFL in concentration range (2–15 µg/mL) using (5 µg/mL) ORN as a divisor at 296 nm in methanol; **b**: Mean Centering of the ratio spectra of ORN in concentration range (3–30 µg/mL) using (4 µg/mL) OFL as a divisor at 315 nm in methanol

The spectra of OFL were divided by the spectrum of ORN (5 µg/mL) to attain the first ratio spectra that were after that mean centered (Fig. 4a). Similarly, the spectra of ORN were divided by the spectrum of OFL (4 µg/mL) and the calculated ratio spectra were mean centered (Fig. 4b). To construct their regression equations, the mean centered values of the 2nd ratio spectra at 296 nm for OFL and 315 nm for ORN, were measured and plotted with respect to the corresponding concentration of each compound as shown in (Fig. S3 and S4).

Calibration curve for OFL (Additional file 1: Fig. S3) and ORN (Additional file 1: Fig. S4) was constructed relating the mean centered values at wave lengths 296 nm and 315, correspondingly and the range of concentration of (2–15 µg/mL) and (3–30 µg/mL), respectively. The regression equations were computed and appear to be:

Y (296 nm) = 0.133X + 0.016, r = 0.9997. (For OFL).

Y (315 nm) = 0.0689X + 0.015, r = 0.9999. (For ORN).

Where Y is mean centered values, X is the drug concentration in µg/mL and r is the correlation coefficient as shown in (Additional file 1: Figs. S3 and S4). The proposed MCR procedure was effectively used to determine OFL and ORN in laboratory-prepared mixtures containing dissimilar proportions of ORN and OFL.

### Continuous wavelet transform of ratio spectra (CWT)

**Fig. 5:**
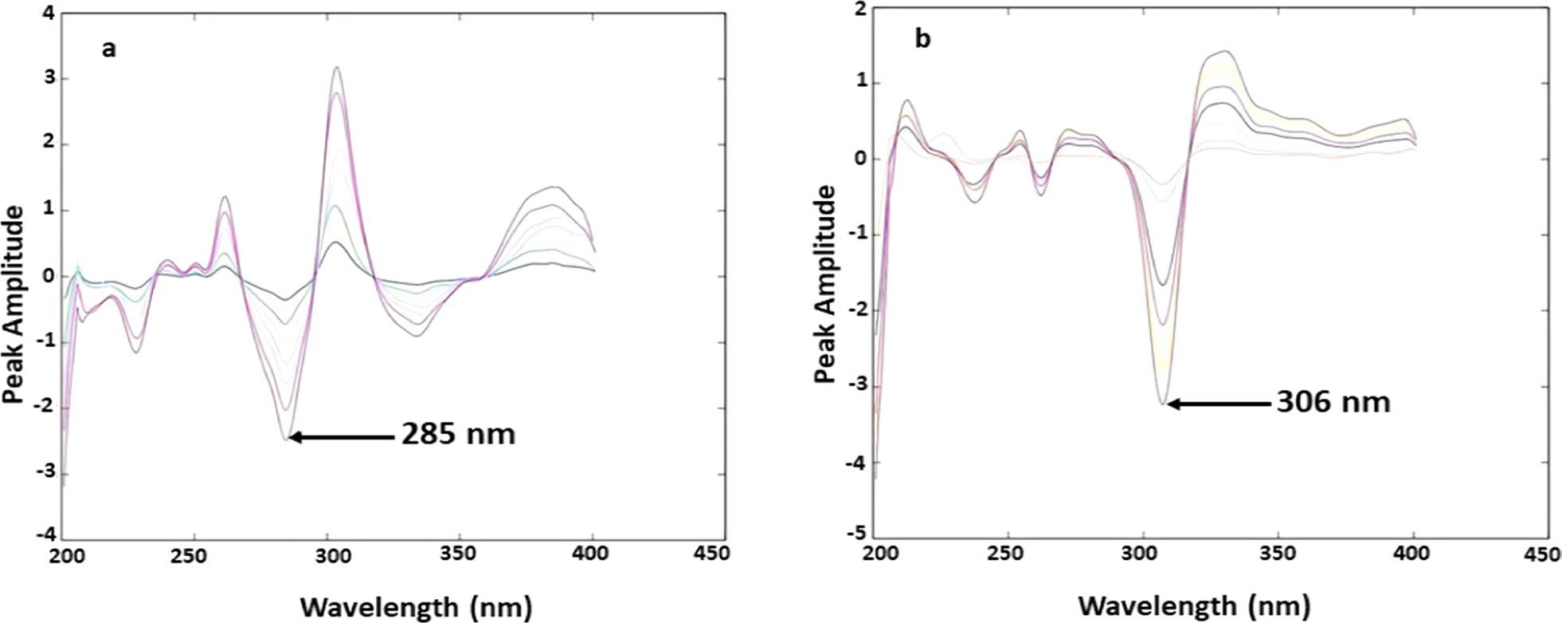
**a**: Wavelet application on the ratio spectra of OFL in concentration range (2–15 µg/mL) using (5 µg/mL) ORN as a divisor at 285 nm in methanol; **b**: Wavelet application on the ratio spectra of ORN in concentration range (3–30 µg/mL) using (4 µg/mL) OFL as a divisor at 306 nm in methanol

To attain the ratio spectra, the absorption spectra of OFL were divided by the absorption spectra of ORN (5 µg/mL) as a divisor. The obtained ratio spectra were used to generate wavelet coefficients using the biorthogonal (bior 2.4) family with [scale value (a) = 25], as shown in (Fig. 5a). Similarly, the recorded spectra of ORN were divided by the spectrum of OFL (4 µg/mL), and the resulting ratio spectra were used to generate wavelet coefficients using the bior 2.4 family with [scale value (a) = 25], as shown in (Fig. 5b). As shown in (Fig. S5 and S6), the amplitudes of these coefficients calculated by CWT at 285 and 306 nm for OFL and ORN.

Calibration curves for OFL (Additional file 1: Fig. S5) and ORN (Additional file 1: Fig. S6) were constructed relating amplitudes of the coefficients at wave lengths 285 nm and 306, correspondingly and the concentration ranges of (2–15 µg/mL) and (3–30 µg/mL), respectively. The regression equations were computed to be:

Y (296 nm) = 0.1636X + 0.021, r = 0.9997. (For OFL).

Y (315 nm) = 0.1083X + 0.0262, r = 0.9998. (For ORN).

Where Y is amplitudes of the coefficients, X is the drug concentration in µg/mL and r is the correlation coefficient as shown in (Additional file 1: Figs. S5, S6). The proposed continuous wavelet of ratio spectra procedure was effectively used to determine OFL and ORN in laboratory-prepared mixtures containing varying proportions of ORN and OFL.

The validity of the developed spectrophotometric techniques was assessed as stated by guidelines of ICH as summarized in (Table 1). From the validation results, it might be concluded that the developed spectrophotometric techniques are precise, accurate and specific over the quantified ranges of concentration. The specificity of the developed spectrophotometric techniques was demonstrated by applying the methods on laboratory prepared mixtures containing varying proportions of the two drugs and reasonable results were found demonstrating the methods' high selectivity as shown in (Table 2).

**Table 1:** Validation parameters of the developed spectrophotometric techniques to determine of Ofloxacin and Ornidazole

Parameters	Ratio Difference		Mean Centering		Continuous Wavelet Transform	
	OFL	ORN	OFL	ORN	OFL	ORN
Range (µg/mL)	(2–15)	(3–30)	(2–15)	(3–30)	(2–15)	(3–30)
Linearity Regression equation	Y = 0.3361 × + 0.112	Y = 0.1751 × + 0.0139	Y = 0.133 × + 0.016	Y = 0.0691 × + 0.0105	Y = 0.1636X + 0.021	Y = 0.1083X + 0.0262
Slope^a^	0.3361	0.1751	0.133	0.0691	0.1636	0.1083
Intercept^a^	0.112	0.0139	0.016	0.0105	0.021	0.0262
Correlation coefficient^a^	0.9999	0.9998	0.9997	0.9999	0.9997	0.9998
Accuracy^b^ (mean)	99.91 ± 0.55	99.86 ± 0.91	99.74 ± 0.86	100.29 ± 0.63	100.14 ± 1.20	99.73 ± 0.82
Specificity^c^	101.10 ± 0.89	99.97 ± 0.69	100.56 ± 0.86	100.77 ± 0.55	100.99 ± 1.07	101.39 ± 0.94
LOQ (µg/mL)^d^	0.98	2.66	1.73	2.30	1.26	2.66
LOD (µg/mL)^e^	0.32	0.87	0.57	0.75	0.74	0.88
Precision (± RSD %)						
a- Intermediate precision^f^	100.01 ± 0.72	100.40 ± 1.08	99.17 ± 0.92	100.43 ± 0.75	99.45 ± 1.21	100.29 ± 0.93
b- Repeatability^g^	100.65 ± 0.98	99.75 ± 1.05	99.56 ± 0.58	100.07 ± 0.64	99.95 ± 1.11	100.89 ± 0.95

^a^Mean of 3 determinations

^b^The average of 3 dissimilar concentrations of ORN and OFL

^c^Recovery of dissimilar laboratory prepared mixtures containing varying proportions of ORN and OFL

^d^Limit of quantitation is calculated

^e^Limit of detection is calculated

^f^Interday precision (the RSD of 3 dissimilar concentrations (6, 8, 10 µg/mL for OFL) and (10, 15, 20 for ORN µg/mL) 3 replicates each, on 3 following days

^g^Intraday precision (the RSD of 3 dissimilar concentrations (6, 8, 10 µg/mL for OFL) and (10, 15, 20 µg/mL) for ORN) 3 replicates each, within the same day

**Table 2:** Determination of Ofloxacin and Ornidazole in laboratory prepared mixtures by the developed spectrophotometric techniques

Mix ratios	OFL: ORN (µg/mL)	Ratio difference		Mean centering		Continuous wavelet transform	
		OFL	ORN	OFL	ORN	OFL	ORN
		*Recovery%	*Recovery%	*Recovery%	*Recovery%	*Recovery%	*Recovery%
1:1	10:10	101.93	100.01	101.80	101.23	101.10	101.92
1:2	10:20	100.53	99.83	100.75	100.54	102.02	101.75
2:1	10:5	99.61	99.38	99.54	101.15	99.81	101.35
1:2.5	10:25	101.33	100.97	100.30	99.76	101.40	101.71
3:1	15:5	101.65	100.52	99.94	101.15	102.03	102.08
1:3	10:30	101.78	99.10	101.05	100.79	99.57	99.53
Mean ± RSD%		101.14 ± 0.88	99.95 ± 0.69	100.56 ± 0.81	100.77 ± 0.56	100.99 ± 1.06	101.39 ± 0.94

^*^Mean of three determinations

Shaded raw: (The ratio of ORN and OFL in their dosage form ORNI-O^™^ tablet)

The developed spectrophotometric techniques were effectively used to analyze the two drugs in their dosage form and applying the standard addition method evaluated the validity of the techniques. The results are displayed in (Table 3). Statistical assessment of the results attained by the developed methods [36, 37] and the reported method by applying the potentiometric method [36] presented that there is not any statistically significance difference as represented in (Table 4).

**Table 3:** Determination of Ofloxacin and Ornidazole in ORNI-O^™^ tablet by the developed spectrophotometric techniques and application of standard addition technique

Marketed drug **(ORNI-O™ tablet)	*Found % ± SD		Standard Addition Technique						*Recovery % of Standard Added	
	OFL	ORN	Conc. (μg/mL)							
			OFL			ORN			OFL	ORN
			Claimed	Standard added	Standard found	Claimed	Standard added	Standard found		
Ratio difference spectrophotometric method	101.10 ± 0.89	101.81 ± 1.14	5	10	10.14	12.5	10	10.12	101.38	101.16
			5	5	5.08	12.5	15	15.40	101.60	102.69
			5	3	2.9	12.5	5	5.12	99.92	102.49
Mean ± SD									100.97 ± 0.91	102.11 ± 0.82
Mean centering method	101.05 ± 0.74	100.98 ± 0.76	5	10	10.06	12.5	10	10.06	100.67	100.68
			5	5	4.99	12.5	15	14.84	99.84	99.96
			5	3	2.98	12.5	5	4.98	99.49	99.78
Mean ± SD									100.01 ± 0.60	99.81 ± 0.86
Continuous wavelet transform	100.59 ± 0.93	101.67 ± 0.42	5	10	9.87	12.5	10	10.15	98.77	101.57
			5	5	4.96	12.5	15	14.86	99.26	99.11
			5	3	3.03	12.5	5	4.98	100.85	99.73
Mean ± SD									99.63 ± 1.08	100.13 ± 1.28

^*^ Mean of 3 determinations

^**^ Batch no. ALT19317

**Table 4:** Statistical comparison of the outcomes attained by the developed technique and the official technique for OFL and ORN determination in the pure powder form

Parameters	RD		MCR		CWT		Official Method^a^	Reported Method^b^
	OFL	ORN	OFL	ORN	OFL	ORN	OFL	ORN
Mean	99.91	99.86	99.74	100.29	100.14	99.73	100.30	99.74
SD	0.55	0.91	0.86	0.66	1.20	1.03	0.85	0.87
N	7	7	7	7	7	7	6	5
Variance	0.31	0.84	0.74	0.45	1.45	1.07	0.72	0.76
F-test^c^	2.38 (4.95)	1.10 (6.16)	1.03 (4.95)	1.69 (6.16)	1.99 (4.95)	1.40 (6.16)		
Student’s t-test^c^	0.97 (2.20)	0.23 (2.22)	1.15 (2.20)	1.25 (2.22)	0.26 (2.20)	0.15 (2.22)		

^a^Potentiometric method [36]

^b^Reported method [37]

^c^Numbers among bows show F and t tabulated at P 0.05

The obtained results prove that the developed techniques can successfully determine OFL and ORN in their formulations and bulk powder.

### Greenness evaluation

The greenness of the method was investigated using the green analytical evaluation tools such as the “Analytical Eco-Scale”, the “National Environmental Method Index” (NEMI) and the “Green Analytical Procedure Index” (GAPI), This evaluation tools are presented in Table 5.

**Table 5:** Greenness evaluation of the developed and reported spectrophotometric approaches to determine Ofloxacin and Ornidazole by Analytical Eco-scale and NEMI

	Analytical Eco-Scale^a^	Penalty points (pp)	NEMI Pictogram^a^	GAPI
Developed spectrophotometric method				
Reagents	Methanol	4		
Instruments (UV-spectrophotometry)	-Energy (< 1.5 kWh per sample -Waste -Occupational hazards	1 8 0		
Total pp		13		
Eco-scale		87		
Reported method [16]				
Reagents	-0.1 N HCl -Phosphate buffer PH 6.8 - Phosphate buffer PH 7.4	4 0 0		
Instruments (UV-spectrophotometry)	-Energy (< 1.5 kWh per sample -Waste -Occupational hazards	1 8 3		
Total pp		16		
Eco-scale		84		
Reported method [18]				
Reagents	0.1 N NaOH 0.1N HCl	4 4		
Instruments (UV-spectrophotometry)	-Energy (< 1.5 kWh per sample -Waste -Occupational hazards	1 8 3		
Total pp		20		
Eco-scale		80		
Reported method [17]				
Reagents	2 M sodium benzoate solution	6		
Instruments (UV-spectrophotometry)	-Energy (< 1.5 kWh per sample -Waste -Occupational hazards	1 8 3		
Total pp		18		
Eco-scale		82		
Reported method [19]				
Reagents	-0.1 N HCl	6		
Instruments (UV-spectrophotometry)	-Energy (< 1.5 kWh per sample -Waste -Occupational hazards	1 8 3		
Total pp		18		
Eco-scale		82		

^a^greenness assessment tools

NEMI is considered one of the first qualitative methods to appraise that the analytical methods are green. NEMI assesses the greenness of the method using a pictogram alienated into four quarters [38]. These quarters signify; PBT (bio accumulative, toxic and persistent) Waste, Corrosive and Hazardous. The corresponding quarter is green shaded, if the chemicals used aren’t classified as PBT via the EPA-TRI [39], the reagents used aren’t harmful hence are not recorded on the TRI list [40], if the medium pH is among 2 and 12 and if the waste produced is fewer than 50 g. We formed the NEMI pictograms for the developed and the four reported techniques (Table 5). From the first glance at the pictograms, the proposed method was green as well as the third reported method, meeting three NEMI criteria, with three green shaded quadrants and greener than the other reported methods. The chemicals and solvents used aren’t stated as PBT, but methanol, which is used in the developed technique, is on the TRI hazardous list [40]. The method is non-destructive, and the waste produced is fewer than 50 g. Concerning the reported techniques, they had two unshaded quadrants matching to hazardous and corrosive quarters.

The analytical Eco-Scale tool is one of numerous green metrics used. It’s commonly used due its advantages over the other techniques as it’s the easiest method in calculations. As well, it can indicate different features of the environmental impact of analytical techniques in its evaluation technique [41]. The ESA value can be calculated through subtracting penalty points from 100 points base for any factor in the method, for example waste production, energy consumption, reagent quantity and hazard. The score should be close to 100 to be considered green. The greater the score (near 100), the more environmentally friendly the technique [41, 42]. The ESA values calculated for the developed and reported methods are presented in (Table 5). According to (Table 5), the developed technique has the greatest score of 87, indicating that it is an excellent green analytical technique with an advanced greenness profile than the reported approaches.

The “Green Analytical Procedure Index” (GAPI) [43] is a new semi-quantitative assessment tool which considered to be a combination between NEMI and ESA tools to assess the greenness of the overall analytical procedure. GAPI is an easy assessment tool for the comparison of different methods and selecting the greenest one. It includes 15 parameters about sample preparation and collection, health and safety impact of reagents and compounds used, waste treatment, and instrumentation. Furthermore, GAPI give a detailed analysis for each analytical procedure step. GAPI uses a three-color scale: green, yellow, or red which represent low, medium, and high ecological influence for each step. The green assessment profiles for the proposed method and the other reported methods using the GAPI tool are shown in (Table 5).

## Conclusion

Signal manipulation to resolve overlapped peaks is important task in digital signal processing. The spectra of ORN and OFL are overlapped, the objective of this work is to resolve this overlap using mathematical models. Three models have been investigated: RD, MCR, and CWT. The main advantage for using the RD method is that it is performed at any two wavelengths across the entire ratio spectrum, without any influence from the overlapped component in the amplitude difference at any wavelength couple. MCR method improves the signal and reduces noise. CWT advances signal-to-noise ratio and has numerous existing families that can suit an extensive range of applications, making it one of the most promising new approaches for manipulating ratio spectra. The main challenge in applying mean centering and continuous wavelet of ratio spectra is that they require prior knowledge of mathematical software such as MATLAB. This makes RD the simplest and most preferable method over the other two (MCR and CWT).

The environmental impact of the three developed approaches was evaluated using the "NEMI", the "Analytical Eco-Scale" tools and “GAPI”, and the proposed techniques were eco-friendly when compared to other reported separation methods. Analytical Eco-Scale is the easiest method. NEMI assesses the greenness of the method using a pictogram alienated into four quarters. GAPI is a combination between NEMI and Analytical Eco-Scale. The developed techniques were authorized in accordance with the ICH guidelines, and all outcomes obtained were satisfactory. All techniques were statistically compared to an official method for OFL and a reported method for ORN and the results indicate that there were not any significant differences.

## Supplementary Information

**Additional file 1: Figure S1.** Linearity of peak amplitude of OFL ratio difference spectra to the corresponding concentrations of OFL (2 - 15 µg/mL) at 265.6 and 294.6 nm in Methanol using (5 µg/mL) ORN as a divisor. **Figure S2.** Linearity of peak amplitude of ORN ratio difference spectra to the corresponding concentrations of ORN (3 - 30 µg/mL) at 292 and 315 nm in Methanol using (4 µg/mL) OFL as a divisor. **Figure S3.** Linearity of peak amplitude of OFL mean centering of the ratio spectra in concentration range (2–15 µg/mL) at 296 nm in MeOH using (5 µg/mL) ORN as a divisor. **Figure S4.** Linearity of peak amplitude of ORN mean centering of the ratio spectra in concentration range (3–30 µg/mL) at 315 nm in MeOH using (4 µg/mL) OFL as a divisor. **Figure S5.** Linearity of peak amplitude of OFL continuous wavelet transform spectra in concentration range (2–15 µg/mL) at 285 nm in MeOH using (5 µg/mL) ORNas a divisor. **Figure S6.** Linearity of peak amplitude of ORN continuous wavelet transform spectra in concentration range (3–30 µg/mL) at 306 nm in MeOH using (4 µg/mL) OFL as a divisor.

## Data Availability

The datasets used and/or analysed during the current study are available from the corresponding author on reasonable request.
