# Supplementary material for: Three developed spectrophotometric methods for determination of a mixture of ofloxacin and ornidazole; application of greenness assessment tools
Source: BMC Chem. 2023 Mar 13;17(1):16. doi: 10.1186/s13065-023-00932-3 (PMC10010042; doi:10.1186/s13065-023-00932-3)
Supplement: Supplementary file 1 — Additional file 1: Figure S1. Linearity of peak amplitude of OFL ratio difference spectra to the corresponding concentrations of OFL (2 - 15 µg/mL) at 265.6 and 294.6 nm in Methanol using (5 µg/mL) ORN as a divisor. Figure S2. Linearity of peak amplitude of ORN ratio difference spectra to the corresponding concentrations of ORN (3 - 30 µg/mL) at 292 and 315 nm in Methanol using (4 µg/mL) OFL as a divisor. Figure S3. Linearity of peak amplitude of OFL mean centering of the ratio spectra in concentration range (2–15 µg/mL) at 296 nm in MeOH using (5 µg/mL) ORN as a divisor. Figure S4. Linearity of peak amplitude of ORN mean centering of the ratio spectra in concentration range (3–30 µg/mL) at 315 nm in MeOH using (4 µg/mL) OFL as a divisor. Figure S5. Linearity of peak amplitude of OFL continuous wavelet transform spectra in concentration range (2–15 µg/mL) at 285 nm in MeOH using (5 µg/mL) ORNas a divisor. Figure S6. Linearity of peak amplitude of ORN continuous wavelet transform spectra in concentration range (3–30 µg/mL) at 306 nm in MeOH using (4 µg/mL) OFL as a divisor. [file 13065_2023_932_MOESM1_ESM.docx]

**Additional file**

**Three Developed Spectrophotometric Methods for Determination of a Mixture of Ofloxacin and Ornidazole; Application of Greenness Assessment Tools**

Khadiga M. Kelani ^1,2*^, Asmaa G. Gad ^2^, Yasmin M. Fayez ^1^, Amr M. Mahmoud ^1*^, Ahmed M. Abdel-Raoof ^3^

^1^ Analytical Chemistry Department, Faculty of Pharmacy, Cairo University, El-Kasr El-Aini Street, 11562, Cairo, Egypt

^2^ Analytical Chemistry Department, Faculty of Pharmacy, Modern University for Technology and Information, Cairo, Egypt

^3^ Pharmaceutical Analytical Chemistry Department, Faculty of Pharmacy, Al-Azhar University, 11751, Nasr City, Cairo, Egypt

^*^Corresponding authors email: [khadiga.elkilany@pharma.cu.edu.eg](mailto:khadiga.elkilany@pharma.cu.edu.eg), [amr.bekhet@pharma.cu.edu.eg](mailto:amr.bekhet@pharma.cu.edu.eg)

**Figure S1.** Linearity of peak amplitude of OFL ratio difference spectra to the corresponding concentrations of OFL (2 - 15 µg/mL) at 265.6 and 294.6 nm in Methanol using (5 µg/mL) ORN as a divisor.

**Figure S2.** Linearity of peak amplitude of ORN ratio difference spectra to the corresponding concentrations of ORN (3 - 30 µg/mL) at 292 and 315 nm in Methanol using (4 µg/mL) OFL as a divisor.

**Figure S3.** Linearity of peak amplitude of OFL mean centering of the ratio spectra in concentration range (2–15 µg/mL) at 296 nm in MeOH using (5 µg/mL) ORN as a divisor.

**Figure S4.** Linearity of peak amplitude of ORN mean centering of the ratio spectra in concentration range (3–30 µg/mL) at 315 nm in MeOH using (4 µg/mL) OFL as a divisor.

**Figure S5.** Linearity of peak amplitude of OFL continuous wavelet transform spectra in concentration range (2–15 µg/mL) at 285 nm in MeOH using (5 µg/mL) ORNas a divisor.

**Figure S6.** Linearity of peak amplitude of ORN continuous wavelet transform spectra in concentration range (3–30 µg/mL) at 306 nm in MeOH using (4 µg/mL) OFL as a divisor.
